# Lung cancer organoids for functional precision oncology: from disease modeling to clinical decision support

**DOI:** 10.3389/fonc.2026.1864153

**Published:** 2026-06-30

**Authors:** Wuxuerong Si, Jiayu Zhou, Ming Jiang

**Affiliations:** 1 Center for Genetic Medicine, International School of Medicine and International Institute of Medicine, Zhejiang University, Yiwu, Zhejiang, China; 2 Department of Thoracic Surgery, Sir Run Run Shaw Hospital, School of Medicine, Zhejiang University, Hangzhou, Zhejiang, China

**Keywords:** bioengineering technologies, drug screening, functional precision oncology, lung cancer, patient-derived organoids, tumor microenvironment

## Abstract

Lung cancer is characterized by extensive heterogeneity and the frequent emergence of acquired resistance, posing major obstacles for successful therapy. Traditional preclinical models, such as two-dimensional (2D) cell cultures and animal models, either lack physiological relevance or require timelines that are poorly coordinated with clinical needs. Lung cancer organoids (LCOs) have been recognized as a promising *in vitro* three-dimensional (3D) platform that retain key features of original tumors, including histological architecture, genetic alterations, and intratumoral heterogeneity. In this review, we summarize recent developments in the area from the perspective of functional precision oncology. We first consider why LCOs are especially valuable, yet technically difficult, in lung cancer, with special focus on sample source, culture bias, contamination by normal airway epithelium, and authentication approaches. We then outline the functional applications of LCOs in recapitulating tumor initiation, evolutionary plasticity, treatment-induced adaptation, immune interactions, drug screening, and biobank construction. Building on these applications, we underscore the critical translational bottlenecks that still constrain routine clinical implementation, such as model fidelity, lack of standardization, temporal restrictions, and inadequate microenvironmental complexity. Lastly, we explore how microfluidic systems, biomimetic matrices, 3D bioprinting, and artificial intelligence (AI)-assisted analytics could help convert LCOs from research models into clinically implementable decision-support platforms. Collectively, these advances place LCOs as a core part of emerging functional precision oncology in lung cancer.

## 1 Introduction: lung cancer needs functional precision models

Lung cancer persists as one of the foremost causes of cancer-related mortality globally, with non-small cell lung cancer (NSCLC) representing approximately 85% of all cases (1, 2). Progress in next-generation sequencing (NGS) has facilitated molecular stratification and the generation of targeted therapies against key oncogenic drivers, including EGFR, ALK, KRAS and BRAF (3–8). However, considerable inter- and intra-tumoral heterogeneity, along with the inevitable occurrence of acquired resistance, continues to restrict therapeutic efficacy (3, 9).

These challenges highlight the requirement for preclinical models that faithfully recapitulate patient-specific tumor biology while supporting fast therapeutic evaluation. During the development of functional precision oncology, multiple patient-derived models have been employed to predict drug sensitivity, investigate mechanisms of therapeutic resistance, and support clinical decision-making. These platforms differ substantially in biological fidelity, establishment efficiency, preservation of the tumor microenvironment (TME), scalability, and clinical applicability, with each model possessing distinct strengths and limitations (**Table 1**).

**Table 1:** Comparison of preclinical models for lung cancer.

Evaluation dimension	Traditional 2D cell lines	CRCs	EVTFs/EVTSs	PDXs	PDOs
Establishment timeline	< 1 week	1–2 weeks	1–3 days	2–6 months	2–6 weeks
Heterogeneity & Structural Preservation	Low. Long-term passaging induces clonal selection, phenotypic adaptation, and severe genetic drift.	Low to moderate. Retains polyclonal architecture better than traditional 2D models, but lacks 3D spatial polarity.	Very high. Largely preserves the native tissue architecture and spatial heterogeneity at the time of sampling.	High. Preserves many histopathological and genomic characteristics of the parental tumor, although mouse-specific selective pressures may alter clonal composition over serial passages.	High. Generally retains major driver mutations and key histopathological characteristics of the sampled tumor tissue.
TME Recapitulation	Absent. Completely lacks stromal and immune compartments.	Absent. Dependent on exogenous feeder cells and specific inhibitors during culture.	Very high. Autologously retains functional TILs, CAFs, and ECM.	Low to moderate. Possesses an in vivo 3D environment with vascularization, but undergoes murine stroma replacement.	Low to moderate. Native stromal and immune compartments are usually lost during expansion but can be partially reconstructed through co-culture systems and microfluidic platforms.
Throughput & Standardization Potential	Very high. Highly standardized and seamlessly adaptable to automated high-throughput screening.	High. Rapid expansion allows medium-to-high-throughput drug sensitivity testing with robust reproducibility.	Low. Constrained by the initial biopsy volume; standardization of slice thickness and viability maintenance remains challenging.	Very low. Limited by animal breeding timelines and escalated logistics costs; unsuited for large-scale compound screening.	High. Compatible with 384-well microplate screening; clinical-grade SOPs are steadily being established.
Major Clinical Translation Advantages	Low cost; highly suitable for early-stage mechanistic studies and large-scale primary compound library screening.	Can be rapidly established and scaled from minimal biopsy specimens, meeting the urgent time window required for extensive drug screening.	Fast turnaround time; represents the unique ex vivo model capable of directly evaluating immediate responses to ICIs.	Features a complete in vivo PK/PD framework; widely regarded as a reference in vivo platform for evaluating systemic drug efficacy.	Provides a favorable balance between clinical turnaround time and biological fidelity, making it well suited for functional precision oncology applications.
Core Clinical Translation Limitations	Fails to reflect in vivo 3D drug responses; poor predictive accuracy for clinical efficacy, making it unsuitable for direct patient care decisions.	Lacks complex microenvironmental cross-talk; clonal phenotypic stability following the withdrawal of reprogramming factors requires further validation.	Extremely short ex vivo lifespan (<1 week); unsuited for evaluating long-term drug exposure or mechanisms of acquired resistance.	Protracted establishment time significantly lags behind the critical first-line clinical decision window; typically lacks a humanized immune system.	At risk of normal airway epithelial overgrowth during primary culture; reconstruction of the stromal/immune compartment escalates system complexity.

Assessments are intended to provide a conceptual framework for comparison and may vary depending on specific culture conditions, tumor subtype, and study design. Qualitative ratings (low, moderate, high) are based on comparative evidence reported across representative studies and should be interpreted as relative rather than absolute assessments. 2D, two-dimensional; 3D, three-dimensional; CRCs, conditionally reprogrammed cells; EVTFs, ex vivo tumor fragments; EVTSs, ex vivo tumor slices; PDXs, patient-derived xenografts; PDO, patient-derived organoid; TILs, tumor-infiltrating lymphocytes; CAFs, cancer-associated fibroblasts; ECM, extracellular matrix; SOPs, standard operating procedures; ICIs, immune checkpoint inhibitors; PK, pharmacokinetics; PD, pharmacodynamics; TME, tumor microenvironment.

Traditional two-dimensional (2D) tumor cell lines remain widely used owing to their low cost, technical simplicity, and compatibility with high-throughput screening. However, prolonged *in vitro* passaging frequently results in clonal selection, genetic drift, and phenotypic adaptation, causing progressive divergence from the biological characteristics of the original patient tumors (10–12). In comparison, conditionally reprogrammed cells, generated through the combination of ROCK inhibition and feeder-cell support, enable rapid expansion of patient-derived tumor cells while partially reducing genetic alterations associated with long-term culture and improving the success rate of primary cell establishment (13). Nevertheless, these cells still represent a fundamentally 2D proliferative system with limited preservation of tissue architecture and spatial tumor heterogeneity. Consequently, although 2D-based models are useful for mechanistic studies and large-scale preliminary drug screening, their predictive value for clinical therapeutic response remains restricted (14).

More recently, tumor explants and ex vivo tissue slice models established from freshly resected patient tumors have increasingly been incorporated into functional precision oncology research. By maintaining intact tumor fragments or thin tissue sections, these systems preserve, to the greatest possible extent, the original cellular composition, spatial organization, and TME components during short-term culture (15). However, their ex vivo viability is generally limited to several days or approximately one week, thereby constraining studies involving prolonged drug exposure or resistance evolution. In addition, variables including tissue slice thickness, oxygen and nutrient diffusion efficiency, and inter-sample standardization may substantially influence experimental reproducibility (16, 17).

Patient-derived xenograft (PDX) models are widely regarded as high-fidelity tumor models because they preserve tumor architecture and certain evolutionary characteristics *in vivo* (18). However, establishment of PDX models typically requires several months, incurs substantial cost, and therefore remains poorly compatible with the limited therapeutic decision-making window encountered in precision oncology (19). Moreover, progressive replacement of human stromal components by murine-derived stroma may compromise accurate recapitulation of the human TME and immune responses (20).

In contrast, lung cancer organoids (LCOs) can generally be established from patient samples within several weeks while retaining key histopathological features, driver genomic alterations, and clonal heterogeneity of the parental tumors. Their three-dimensional (3D) architecture more closely resembles native tumor organization, making them particularly suitable for drug sensitivity testing, investigation of resistance evolution, and development of companion diagnostic strategies (21, 22). Nevertheless, LCOs still face several important limitations, including variable establishment efficiency, overgrowth of normal epithelial cells, and incomplete reconstruction of the tumor TME.

More recently, organ-on-a-chip and microfluidic platforms have further improved the ability to model dynamic TME-associated processes. These systems can integrate vascular shear stress, immune-cell migration, and drug concentration gradients, thereby enabling more physiologically relevant reconstruction of tumor–stroma–immune interactions (23, 24). However, their relatively high technical complexity and lack of standardized workflows currently limit their application largely to experimental research settings, and routine clinical implementation remains premature (23, 25).

Overall, the various models used in functional precision oncology should not be viewed as mutually exclusive, but rather as complementary systems with distinct translational advantages. 2D cell lines are well suited for high-throughput mechanistic screening, PDX models are valuable for long-term *in vivo* validation, and organ-on-a-chip systems provide unique opportunities for investigating dynamic microenvironmental interactions. Within this spectrum, LCOs achieve a comparatively favorable balance among clinical feasibility, biological fidelity, and experimental scalability, positioning them as a particularly promising platform for functional precision oncology.

## 2 LCOs: opportunities, technical challenges, and authentication

### 2.1 LCOs are especially valuable

LCOs are especially promising in lung cancer, since the disease is molecularly subtyped but functionally unpredictable. In numerous instances, genotyping uncovers actionable drivers, but response to treatment is further determined by lineage state, tumor evolution, microenvironmental context, and prior therapeutic exposure. Additionally, advanced lung cancer often supplies only tiny biopsies or fluid samples, which raises the importance of scalable *ex vivo* systems that can be derived from limited material and repeatedly sampled over time.

**Figure 1:**
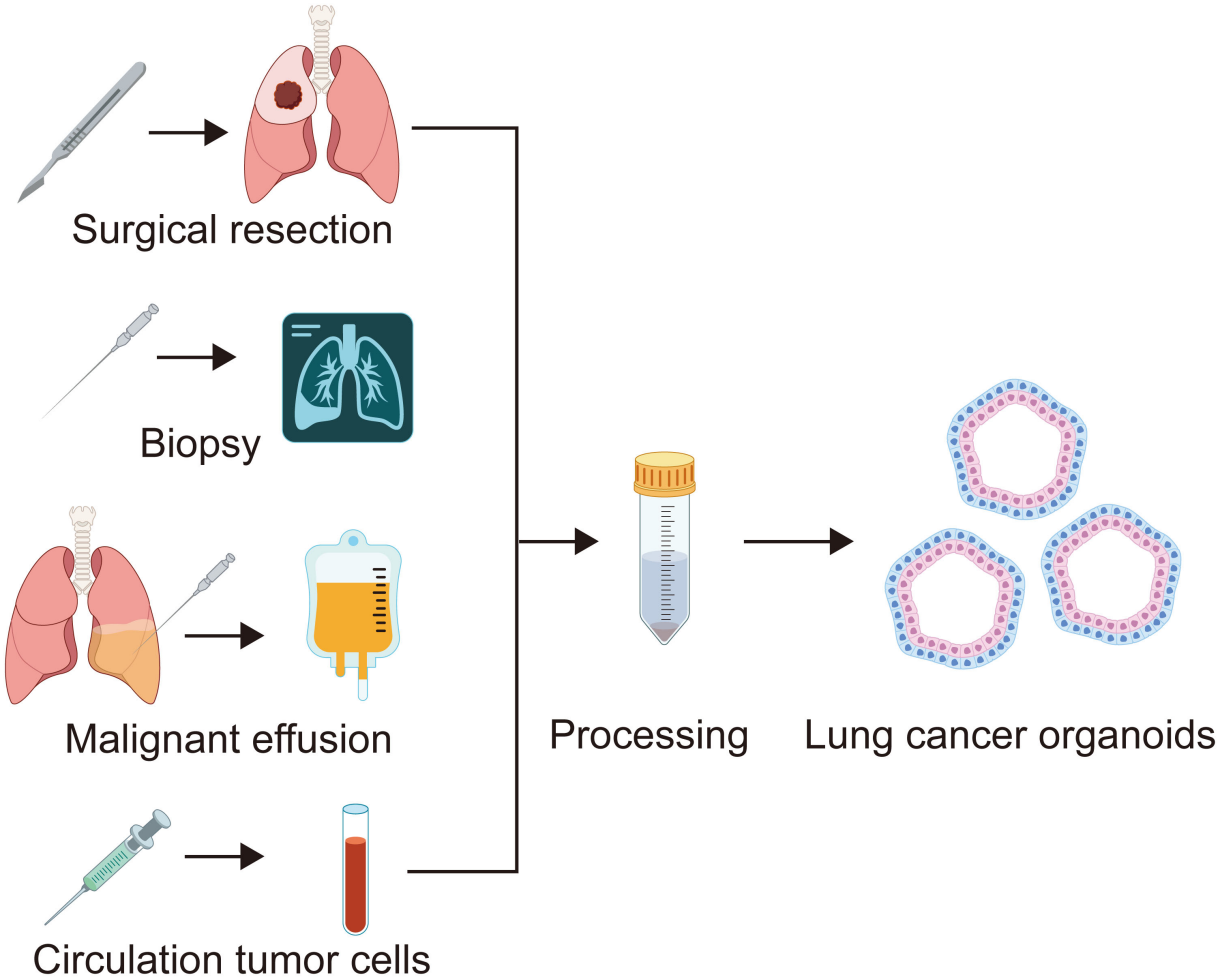
Diverse cellular and clinical sources for the establishment of LCOs. LCOs, lung cancer organoids.

LCOs can be established from multiple types of clinical samples, such as surgical resections, biopsies, malignant effusions, and circulating tumor cells (CTCs) (**Figure 1**) (26). Of these, malignant effusions commonly provide higher establishment success rates, probably owing to higher tumor cell content and less contamination (27). Furthermore, microfluidic-based co-culture systems have supported the creation of organoids from CTCs, delivering a minimally invasive strategy for patient-specific modeling (28).

### 2.2 Technical bottlenecks in LCO generation

Organoids are spontaneously organizing 3D structures derived from pluripotent or adult stem cells that mimic key architectural and functional properties of original tissues (29). A significant milestone occurred in 2009, when the laboratory of Hans Clevers successfully created a mouse intestinal organoid culture system, laying a basis for subsequent research on organoid development and disease modeling (30). Progress in organoid culture systems has supported the establishment of LCOs from patient specimens, representing a physiologically faithful platform for disease modeling. A principal technical difficulty in LCOs establishment is contamination with normal airway epithelial cells, which can outgrow tumor cells in culture. To tackle this problem, selective culture methods have been created. For example, the addition of Nutlin-3a leverages the differential p53 status between normal and tumor cells, enabling selective elimination of wild-type epithelial cells and increasing tumor purity (31).

Both extracellular matrix (ECM) constituents and biopsy source critically affect organoid establishment efficiency and quality. Matrigel and basement membrane extract (BME) stay broadly used but are affected by batch variability and undefined composition, hindering reproducibility and clinical translation. Emerging substitutes, such as synthetic hydrogels and decellularized ECM, give enhanced control over mechanical and biochemical features, thereby increasing model consistency (32).

In summary, the successful generation of lung cancer organoids depends on more than simply establishing viable 3D cultures, and it requires careful control of tumor cell selection, matrix composition, sampling strategy, and culture conditions to preserve the biological features of the original tumor. Therefore, optimized culture protocols, such as selective media, engineered matrices, and varied sampling approaches, are necessary for elevating the efficiency, reproducibility, and translational feasibility of LCOs (33).

### 2.3 Authentication and quality control for LCOs

Precise validation of LCOs is vital for ensuring their reliability in downstream applications. A major disadvantage comes from the frequent contamination of organoid cultures by normal airway-derived epithelial cells, which cannot be reliably discriminated from tumor organoids through morphology alone. To solve this problem, a combination of histological and genomic approaches is needed. Immunohistochemical (IHC) analysis, such as p63 staining, can aid in distinguishing normal airway organoids from tumor-derived structures, though it is not adequate as a sole method (34).

At the molecular level, genomic profiling provides a more robust strategy for validation. Copy number variation (CNV) analysis is routinely regarded as a definitive standard for confirming tumor origin, whereas whole-exome sequencing (WES) and RNA sequencing (RNA-seq) further allow comprehensive characterization of genetic and transcriptional properties. Well-qualified LCOs have been demonstrated to preserve the mutational repertoire and molecular characteristics of the original tumors with good correlation (27, 35).

Besides, organoids generated from various sources, such as CTCs, have demonstrated the capability to retain clinically relevant genomic alterations and therapeutic response profiles, further supporting their applicability in precision oncology (28).

In conclusion, the union of morphological assessment with multi-omics validation is essential for guaranteeing the fidelity, reproducibility, and translational relevance of LCO models. Moreover, standardized authentication workflows are particularly important when LCOs are used for precision oncology. Establishing rigorous and reproducible validation criteria will be critical for improving confidence in LCO-based studies and facilitating their broader application in drug screening, biomarker discovery, and individualized treatment selection.

## 3 Functional applications of LCOs

### 3.1 Tumor initiation, evolution, and lineage plasticity

LCOs are capable of preserving the genetic and phenotypic characteristics of parental tumors while supporting long-term culture and dynamic experimental manipulation, thereby providing a physiologically relevant platform for investigating lung tumorigenesis and progression. When integrated with gene-editing technologies and single-cell multi-omics approaches, LCOs further enable systematic dissection of early oncogenic events and tumor evolutionary processes within a context that more closely resembles the native tumor environment.

Current organoid-based studies of tumor initiation mainly focus on the activation of cell-intrinsic oncogenic signaling pathways. For instance, activation of oncogenic KRAS in alveolar type II (AT2) progenitor cells induces lineage reprogramming characterized by downregulation of differentiation-associated genes (36). Similarly, human induced pluripotent stem cell (hiPSC)-derived organoid models have successfully recapitulated HER2-driven malignant transformation (37). Beyond canonical oncogenic mutations, organoid systems have also highlighted the importance of spatial organization in tumor development, including oncogenic signaling mediated through liquid–liquid phase separation (LLPS) of fusion proteins (38).

In modeling tumor evolution and organ-specific metastasis, LCOs combined with *in vivo* transplantation systems provide distinct advantages for dynamically investigating clonal evolution, metastatic dissemination, and the emergence of therapeutic resistance. For example, by establishing patient-derived primary and metastatic organoid models, investigators were able to reconstruct resistance-associated clonal evolutionary trajectories of KRAS G12C-mutant tumors under sotorasib treatment (39). In clinically challenging settings such as bone metastasis, patient-derived organoid (PDO) models have likewise been employed to uncover the role of SLC2A3-mediated metabolic reprogramming in regulating p53 lactylation, while also demonstrating the potential synergistic efficacy of Paris saponin VII combined with anti-PD-1 therapy (40). In small cell lung cancer (SCLC), orthotopic transplantation of gene-edited organoids further revealed that loss of KMT2C promotes distant metastasis through histone and DNA hypomethylation, underscoring a critical epigenetic mechanism underlying metastatic progression (41).

Moreover, organoid models have demonstrated that tumors can undergo phenotypic transitions under selective pressure, reflecting the phenomenon of lineage plasticity (42). This plasticity represents not only a fundamental characteristic of tumor evolution but also a major mechanistic basis for clinical drug resistance (43). However, owing to the absence of long-term complex stromal support and sustained chronic therapeutic pressure, current short-term *in vitro* systems remain unable to fully recapitulate the extensive spectrum of lineage transitions observed clinically (44). Consequently, future studies will need to move beyond single-dimensional oncogenic events toward more integrated systems-level investigations that incorporate biomechanical regulation, complex microenvironmental components, and long-term chronic survival stress.

### 3.2 Drug resistance and therapy-induced adaptation

LCOs enable *in vitro* modeling of tumor evolution under therapeutic pressure and therefore provide a valuable platform for dissecting acquired resistance mechanisms and identifying potential reversal strategies.

Under the persistent selective pressure imposed by targeted therapies, tumor phenotypic evolution exhibits remarkable plasticity. Organoid-based studies have uncovered multiple non-genetic mechanisms underlying therapeutic resistance. For example, EGFR-mutant tumors may undergo basal-like transitions following loss of the lineage-defining transcription factor NKX2-1, ultimately resulting in broad resistance to EGFR-TKIs (45). Similarly, adenosquamous transformation (AST) has also been observed during KRAS-targeted therapy, in which upregulation of KRT6A not only promotes adaptive resistance but additionally serves as a predictive biomarker for response to adagrasib (46). Other studies have further identified loss of the histone methyltransferase KMT2D as an epigenetic driver of AST (47).

At the level of molecular regulation, LCOs provide a highly physiologically relevant platform for identifying potential sensitizing targets, particularly in the context of resistance to specific targeted agents. It has been reported that the RNA-binding protein RBM15 promotes osimertinib resistance by enhancing m^6A modification of SPOCK1 mRNA, whereas early targeting of RBM15 in organoid models can effectively prolong the therapeutic window of TKIs (48). Likewise, the demethylase FTO has been shown to drive tumor progression through the IGFBP3/AKT signaling pathway (49). In addition, through genome-wide CRISPR screening combined with organoid-based functional assays, the efflux transporter ABCC1 was identified as a critical mediator of resistance to KRAS^G12C inhibitors, and co-treatment with Src inhibitors effectively overcame this resistance barrier (50).

With respect to chemotherapy resistance and evasion of programmed cell death, LCOs have further highlighted the profound metabolic adaptability of tumor cells. In highly recurrent SCLC, systemic dysregulation of the pyrimidine synthesis pathway has been demonstrated to represent a major determinant of chemotherapy failure (51). Notably, certain metabolic enzymes also exhibit noncanonical functions beyond their traditional metabolic roles. For example, glutamine synthetase (GS) can sustain mitotic progression independently of its catalytic activity through interaction with NUP88 (52). Furthermore, disruption of ATP7A-mediated copper homeostasis using copper ionophores has provided a novel strategy for inducing cuproptosis in LCOs, thereby offering a potential therapeutic approach for refractory lung cancer (53). Tumor cells may additionally evade programmed cell death through remodeling of the TME. Using PDOs, Su et al. demonstrated that chemotherapy-induced stress activates the β5-integrin/Src/STAT3 signaling axis, leading to upregulation of ceramidase (ASAH2) and suppression of chemotherapy-induced pyroptosis (54).

Collectively, findings derived from LCO models suggest that therapeutic resistance is far more complex than the acquisition of isolated target-gene mutations, instead representing a global adaptive process involving lineage plasticity, post-transcriptional regulation, and metabolic compensation. Future studies utilizing LCO-based resistance models will likely move beyond single-mechanism investigations toward systems-level approaches integrating microenvironmental stress, physiologically optimized culture conditions, and longitudinal multi-omics profiling.

### 3.3 Immune co-culture and immunotherapy assessment

The complexity of the TME plays a central role in shaping responses to immunotherapy. Conventional organoid systems, owing to their lack of native stromal and immune components, have long been limited in their ability to evaluate immunotherapeutic efficacy. In recent years, advances in co-culture strategies and engineered matrices have progressively transformed LCOs from simple tumor-cell models into platforms capable of partially reconstructing the immune ecosystem (55). Nevertheless, substantial differences remain among various immune-organoid systems with regard to culture duration, preservation of immune compartments, and the specific biological questions they are suited to address.

Short-term immune co-culture systems rapidly evaluate tumor–immune interactions through the exogenous introduction of immune cells into established organoids. Such models enable fast assessment of immune-mediated cytotoxicity and are therefore particularly useful for functional evaluation of chimeric antigen receptor (CAR)-T cells, natural killer (NK) cells, and immune checkpoint inhibitors (ICIs), while also facilitating investigation of specific immune escape mechanisms (56). For example, Palade et al. co-cultured patient-derived NSCLC organoids with NK cell-derived extracellular vesicles (NKEVs) and demonstrated that NKEVs reduced tumor cell viability while enhancing sensitivity to cisplatin. When combined with nivolumab, NKEVs additionally altered the immune-cell landscape, characterized by reduced CD4^+^ T cells and increased CD56^+^ NK cells (57). The gel-liquid interface (GLI) co-culture system established by Li et al. further enhanced direct interactions between immune cells and tumor cells, enabling prediction of patient responses to anti-PD-1 therapy and identification of circulating tumor-reactive T cells with an effector-memory phenotype (GNLY^+^CD44^+^CD9^+^) as potential biomarkers (58). In addition, co-culture of organoids with peripheral blood mononuclear cells (PBMCs) has been shown to effectively expand tumor-reactive T cells, achieving success rates of 33%-50% in NSCLC (59, 60).

Despite these advantages, major limitations remain in short-term co-culture systems. Most immune populations are introduced exogenously after organoid establishment and therefore lack the native spatial organization and long-term selective pressures present within authentic TMEs, making it difficult to preserve physiological cellular hierarchies. Furthermore, PBMCs or expanded T cells are not fully equivalent to tumor-resident infiltrating immune populations, since their T-cell receptor (TCR) repertoires and exhaustion states may already have been altered during ex vivo manipulation. These systems also generally support T-cell viability only over relatively short periods, limiting their capacity to model chronic antigen stimulation, immune editing, and long-term resistance evolution (61). More importantly, they frequently lack a complete myeloid compartment, including tumor-associated macrophages (TAMs), dendritic cells (DCs), and neutrophils, thereby failing to comprehensively recapitulate the immunosuppressive network within the TME (62).

In contrast, air-liquid interface (ALI) and partially native tissue-preserving culture systems place greater emphasis on long-term maintenance of original tumor architecture in an effort to retain a more complete immune ecosystem (63). Using an ALI-based approach, Neal et al. established PDOs from more than 100 human biopsy samples and syngeneic immunocompetent mouse tumors while preserving both tumor epithelium and endogenous immune populations. Single-cell analyses demonstrated that tumor-infiltrating lymphocytes (TILs) within these organoids accurately retained the TCR repertoire of the parental tumors (64). Such systems typically avoid complete tissue dissociation and instead maintain tumor fragments, thereby preserving, at least transiently, the native spatial relationships among tumor cells, stromal cells, and immune components (65).

However, even ALI-based systems remain unable to fully reconstruct a stable and durable immune ecosystem. Endogenous immune populations progressively decline during prolonged culture, with myeloid cells and B cells being particularly difficult to maintain long term. In addition, the absence of a circulatory system prevents accurate modeling of immune-cell recruitment, cytokine gradients, and lymphatic drainage, thereby limiting faithful simulation of immune-cell infiltration dynamics and immunotherapy-associated toxicities (66). Even when LCO-based immune co-culture systems are employed, responses to ICIs remain difficult to predict accurately because clinical benefit depends not only on intrinsic tumor-cell properties but also on dynamic processes within the TME, including T-cell exhaustion, myeloid-mediated suppression, spatial heterogeneity, and systemic immune status, all of which are challenging to sustain *in vitro* (67–70). Furthermore, patient-specific TCR repertoires, HLA backgrounds, and patterns of clonal evolution are difficult to preserve comprehensively under ex vivo conditions (71, 72).

### 3.4 Drug screening and personalized therapy

Drug sensitivity assessment is currently regarded as one of the most translationally promising applications of LCOs. By recapitulating individualized responses to specific therapies *in vitro*, LCOs provide an important framework for bridging basic cancer research with functional precision oncology.

With respect to prediction of clinical therapeutic response, LCOs have demonstrated encouraging concordance across multiple studies. A large-scale retrospective analysis reported that LCOs derived from malignant serous effusions (MSEs) achieved an 83.3% concordance rate in predicting responses to chemotherapy and targeted therapies (27). In patients harboring complex genotypes, such as EGFR 19del combined with BRAF G464A mutations or the rare EGFR L747P variant, *in vitro* drug responses observed in LCOs were also found to reflect, to a certain extent, the actual clinical efficacy of dabrafenib plus trametinib or afatinib treatment, thereby providing valuable guidance for individualized therapeutic decision-making in the setting of complex mutational landscapes (35). Nevertheless, current clinical evidence for LCO-guided therapy still relies predominantly on feasibility studies, small patient cohorts, and concordance analyses of drug sensitivity, while large-scale, multicenter prospective randomized trials using overall survival (OS) as a primary endpoint remain lacking (73, 74). In addition, lung cancer is characterized by marked spatial and temporal heterogeneity, meaning that LCOs established from single-site biopsy specimens may themselves introduce substantial sampling bias (75). From the perspective of clinical timeliness, although several optimized workflows have shortened the interval required for LCO-guided treatment evaluation to approximately 10 days, many standard organoid establishment and drug screening pipelines still require several weeks to complete. Consequently, for patients with rapidly progressing advanced lung cancer who require immediate therapeutic intervention, the feasibility of LCOs as real-time clinical decision-support tools remains constrained (76, 77).

In the context of modeling rare mutations and developing novel therapeutic agents, PDOs help alleviate the limitation of restricted clinical tissue availability. Under molecular backgrounds such as MET exon 14 skipping mutations and EGFR exon 20 insertion mutations, LCOs have been employed to evaluate the anti-tumor activity of the multikinase inhibitor ensartinib and the bispecific antibody amivantamab, with amivantamab demonstrating superior efficacy compared with cetuximab or poziotinib (78, 79). In SCLC, organoid models have further identified YES1 as a potentially actionable oncogenic driver and recognized RepID protein as a biomarker for screening cullin-RING ligase (CRL)-targeted therapies in neuroendocrine tumors (80, 81). At present, however, most investigations remain confined to single-center laboratory studies. Considerable variability persists among laboratories regarding organoid culture media formulations, batch control of matrix hydrogels, concentration-gradient design for drug sensitivity testing, and criteria used for evaluating cellular viability. Such inconsistencies directly affect inter-study reproducibility and hinder the establishment of globally accepted clinical cutoff values for drug sensitivity assessment (82, 83).

Beyond conventional therapeutic testing, the application scope of LCOs has recently expanded into emerging areas including drug engineering, such as CD155-targeted nanobody-conjugated liposomes and mechanistic studies of fangchinoline, as well as innovative immunotherapeutic strategies, including probiotic-inspired nanosystems for remodeling “cold” TMEs and integration with quantitative systems pharmacology (QSP) models for individualized dose prediction (84–87). Collectively, these developments further underscore the value of LCOs as a highly scalable translational platform for drug discovery and precision oncology.

### 3.5 Biobanks and longitudinal modeling

LCOs, owing to their capacity for long-term ex vivo expansion while largely preserving the genomic and phenotypic characteristics of parental tumors, have emerged as an important platform for the construction of living lung cancer biobanks.

By integrating tumor purity assessment with optimized culture protocols, investigators have successfully established organoid collections representing major lung cancer subtypes, including adenocarcinoma, squamous cell carcinoma, SCLC, and large cell carcinoma, with relatively high establishment success rates (88, 89). These biobanks provide substantial value in capturing tumor spatial heterogeneity, supporting large-scale molecular stratification, and facilitating anti-cancer drug development. For example, in SCLC, organoid models retaining hallmark features such as co-loss of TP53 and RB1 have contributed to the development of subtype-oriented precision therapeutic strategies based on transcriptomic classification (90). In addition, the application of minimally invasive sampling approaches, including organoids derived from sputum specimens or CTCs, has further broadened the coverage of organoid biobanks and provided a foundation for longitudinal monitoring of tumor evolution (89). Compared with conventional patient-derived xenograft (PDX) models, organoid platforms possess clear advantages in establishment time and high-throughput scalability, making them particularly useful for uncovering growth dependencies in rare tumor subtypes such as neuroendocrine malignancies (91, 92).

Comparative transcriptomic analyses have further demonstrated that the growth-suppressive effects of certain key regulatory factors, including CDC25A, are more pronounced in organoid systems than in traditional 2D cultures, highlighting the unique ability of organoids to capture physiologically relevant tumor biology (93). In SCLC, Choi et al. established patient-derived tumor organoids and identified distinct subpopulations exhibiting differential sensitivity to cisplatin and etoposide. This degree of intratumoral heterogeneity paralleled the clonal diversity observed in patients and corresponded closely with differences in clinical outcomes (94). However, during prolonged ex vivo expansion, organoids frequently undergo selective evolutionary processes. The selective pressure imposed by specific culture media formulations may preferentially enrich particular cellular subpopulations, ultimately resulting in clonal compositions that diverge from those of the original tumors (95). At the same time, LCO biobanks still lack harmonized standards across institutions regarding culture systems, establishment criteria, and molecular characterization workflows. Such substantial inter-laboratory variability makes direct comparison among independently generated biobanks difficult and limits their broader applicability as universal “clinical diagnostic reference” platforms (95, 96).

**Figure 2:**
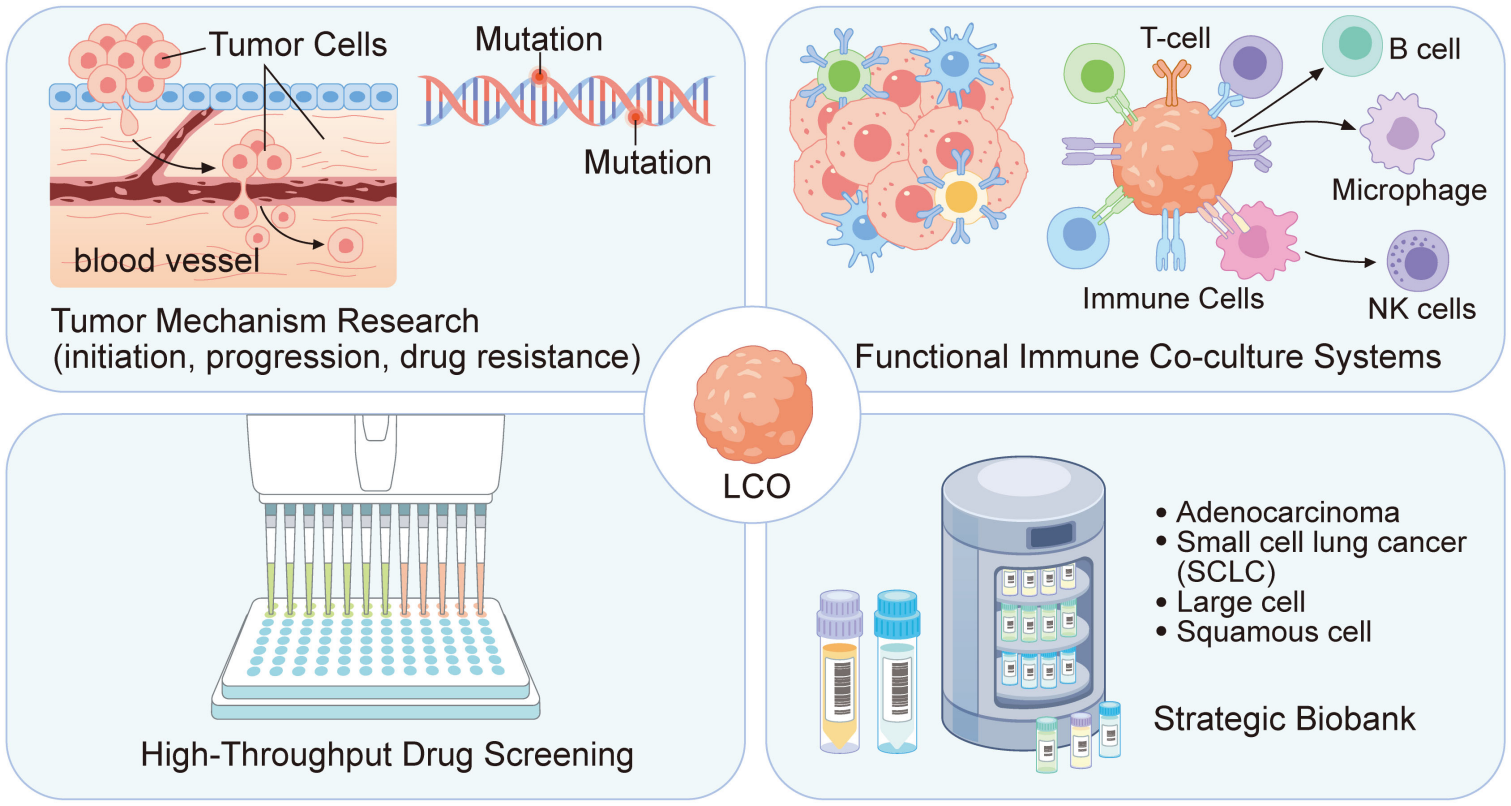
Applications of LCOs in basic research and translational medicine.

Overall, LCO biobanks establish a scalable and multifunctional research platform that serves as an important bridge between fundamental investigation and clinical translation. Through integration of patient-derived models, molecular characterization, and functional drug testing, these platforms are driving functional precision oncology toward increasingly individualized and mechanism-oriented therapeutic strategies (**Figure 2**).

## 4 Engineering and computational strategies to overcome translational barriers

Although LCOs have shown considerable promise in disease modeling, drug screening, and precision medicine, their clinical translation remains constrained by several key limitations, including the lack of standardized culture and quality control protocols, prolonged drug sensitivity testing that fails to meet the demands of rapid clinical decision-making, and the absence of a fully recapitulated TME.

In this context, technological advances have become increasingly critical in addressing these bottlenecks. Instead of acting as mere technical enhancements, microfluidic systems, sophisticated biomaterials, bioprinting strategies, and AI-assisted analytics are progressively being designed as targeted solutions to improve speed, standardization, biological realism, and interpretability.

### 4.1 Microfluidics for speed, control, and throughput

Conventional 3D culture systems often exhibit high batch-to-batch variation and lack essential physical signals from the microenvironment, including fluid shear stress and mechanical forces (97, 98). Microfluidic technology provides an effective engineering solution by permitting precise spatiotemporal regulation of hydrodynamics and metabolite exchange (99, 100). For standardized production, techniques such as droplet microfluidics or 3D-printed custom microwell arrays (e.g., 200 μm agarose wells) enable the reproducible generation of lung cancer assembloids (LCAs) or tumor-stroma co-culture spheroids from limited patient-derived samples. These highly uniform models retain the clonal heterogeneity of the original tumors while faithfully recapitulating stromal cell-mediated drug resistance (101, 102). To accelerate clinical application, integrated microphysiological systems (MPS) and InSMAR chips (inverse colloidal crystal-structured, superhydrophobic microwell arrays) have significantly shortened testing times, allowing drug sensitivity profiling from primary organoids within one week with results that align closely with patient outcomes (103, 104). Furthermore, next-generation microfluidic platforms are advancing toward completely integrated pipelines. For example, Zhang et al. integrated DNA nanoprobes with organoid chips to carry out molecular diagnosis of EGFR mutations and real-time tracking of targeted drug efficacy within hours, substantially extending the scope of organoid-based precision medicine (105).

### 4.2 Biomaterials and 3D bioprinting for structural realism

Although microfluidic platforms offer clear benefits for standardized drug screening, faithfully reconstituting a complex TME *in vitro*, especially one containing functional vascular networks, depends heavily on the combination of 3D bioprinting with advanced biomaterials. Advances in scaffold design provide the required mechanical and biochemical signals to support physiologically relevant cell-cell interactions. For instance, functionalized alginate microbeads have been utilized as biomimetic alveolar scaffolds, supporting co-cultures of SCLC cells and fibroblasts, and underscoring the essential role of stromal paracrine signaling in tumor regrowth following chemotherapy (106). To better mimic the native ECM, decellularized lung ECM (LudECM) has been integrated into bioink formulations as a substitute to Matrigel. When coupled with 3D printing, this approach enables the creation of vascularized models containing LCOs, cancer-associated fibroblasts, and endothelial cells. These systems retain patient-specific genetic profiles while offering perfusable vascular networks that promote organoid growth, providing a physiologically relevant platform for evaluating the pharmacokinetics and pharmacodynamics of intravenously delivered drugs (107). By supplying tumor-specific extracellular matrices, suitable mechanical properties, and accurately controlled geometries, bioprinting allows more faithful reconstruction of the TME. Advanced methods including inkjet and acoustic printing further enhance the flexibility of this strategy, allowing simultaneous deposition of multiple cell types and matrix constituents for the construction of intricate, multicellular tissue platforms (108, 109).

### 4.3 AI-assisted organoid analytics for scalable interpretation

The rapid expansion of artificial intelligence (AI) in oncology is accelerating the transition of organoid-based precision medicine from conventional endpoint measurements toward multidimensional mechanistic investigation. Traditional organoid drug screening has relied heavily on ATP-based luminescence assays, yet this single-dimensional metabolic evaluation often fails to capture dynamic biological responses and may even obscure subtle dose-dependent toxicities induced by certain compounds (110). The integration of deep learning with high-content imaging (HCI) has begun to overcome this limitation by enabling non-invasive, multiparametric, and longitudinal phenotypic profiling (111). For instance, the deep learning-based object detection algorithm Deep-LUMEN can automatically monitor lumen morphology and epithelial polarization in lung spheroids using brightfield imaging, thereby substantially reducing the subjectivity associated with manual assessment of PDO growth (110, 111). In parallel, the large-scale datasets generated from high-throughput experiments provide extensive resources for AI model training, helping alleviate the data scarcity that has historically constrained conventional drug discovery workflows. These developments are further promoting AI-assisted experimental pipelines capable of quantifying cellular states, reconstructing developmental trajectories, and actively classifying molecular phenotypes (112–115).

As the concept of “AI-empowered organoids” continues to evolve, the integration of high-dimensional multi-omics and imaging datasets generated by high-throughput screening (HTS) platforms into AI systems is expanding beyond drug response prediction alone and increasingly contributing to candidate drug design and optimization (116). At the level of clinical drug sensitivity prediction, models such as PharmaFormer have markedly improved predictive accuracy through pretraining on pan-cancer cell line sensitivity datasets followed by fine-tuning using organoid pharmacogenomic data (117). Combined with droplet microfluidics and closed-loop microfluidic platforms, AI-driven image analysis not only shortens the interval between tumor resection and drug sensitivity assessment, but also distinguishes cytotoxic from cytostatic effects while accurately resolving differential drug responses between PDOs and stromal components. Such systems provide a new technical framework for identifying optimal combination regimens, including treatment sequence, dosage, and scheduling (118, 119).

Despite these advances, the integration of AI with organoid technologies still faces several major obstacles. First, AI models depend heavily on large-scale, high-quality datasets, yet studies involving rare diseases or emerging therapeutic targets are frequently constrained by limited data availability, high acquisition costs, and concerns surrounding patient privacy, all of which restrict model generalizability (120, 121). Existing datasets also commonly contain bias, annotation errors, and experimental inconsistencies, factors that can markedly impair predictive performance when models are applied to novel molecular structures or previously unseen target interactions (122). Second, 3D organoid imaging remains technically challenging because optical limitations and tissue thickness hinder acquisition of complete spatial ground-truth annotations. Batch effects introduced by differences in culture conditions and imaging platforms may further generate systematic bias capable of obscuring authentic biological signals (123). In addition, image-based deep learning systems possess inherent vulnerabilities related to imbalanced training datasets and annotation variability, which may reduce performance when models are extended to new patients or unfamiliar tissue types (124). Reproducibility also remains a persistent concern. Deep learning frameworks are highly sensitive to training conditions, while insufficient code sharing and lack of standardized reporting continue to limit cross-laboratory reproducibility of biomedical AI models (125). From a regulatory perspective, AI-assisted diagnosis and drug sensitivity prediction directly involve patient safety. The limited interpretability of black-box models, together with the absence of harmonized validation standards, continues to restrict regulatory confidence in their clinical implementation (122).

Overall, AI-enhanced organoid platforms are developing rapidly and are demonstrating distinct advantages in drug screening, phenotypic profiling, and mechanistic investigation. Nevertheless, achieving clinically actionable personalized medicine will require continued efforts to address challenges involving data harmonization, reproducibility, bias control, and regulatory interpretability. Future studies should prioritize the establishment of multimodal and highly standardized training datasets while promoting deeper integration among AI systems, spatial omics technologies, and clinically annotated datasets. Such advances will be essential for fully realizing the transformative potential of AI in organoid-based precision oncology.

### 4.4 Integrated workflows for functional precision oncology

**Figure 3:**
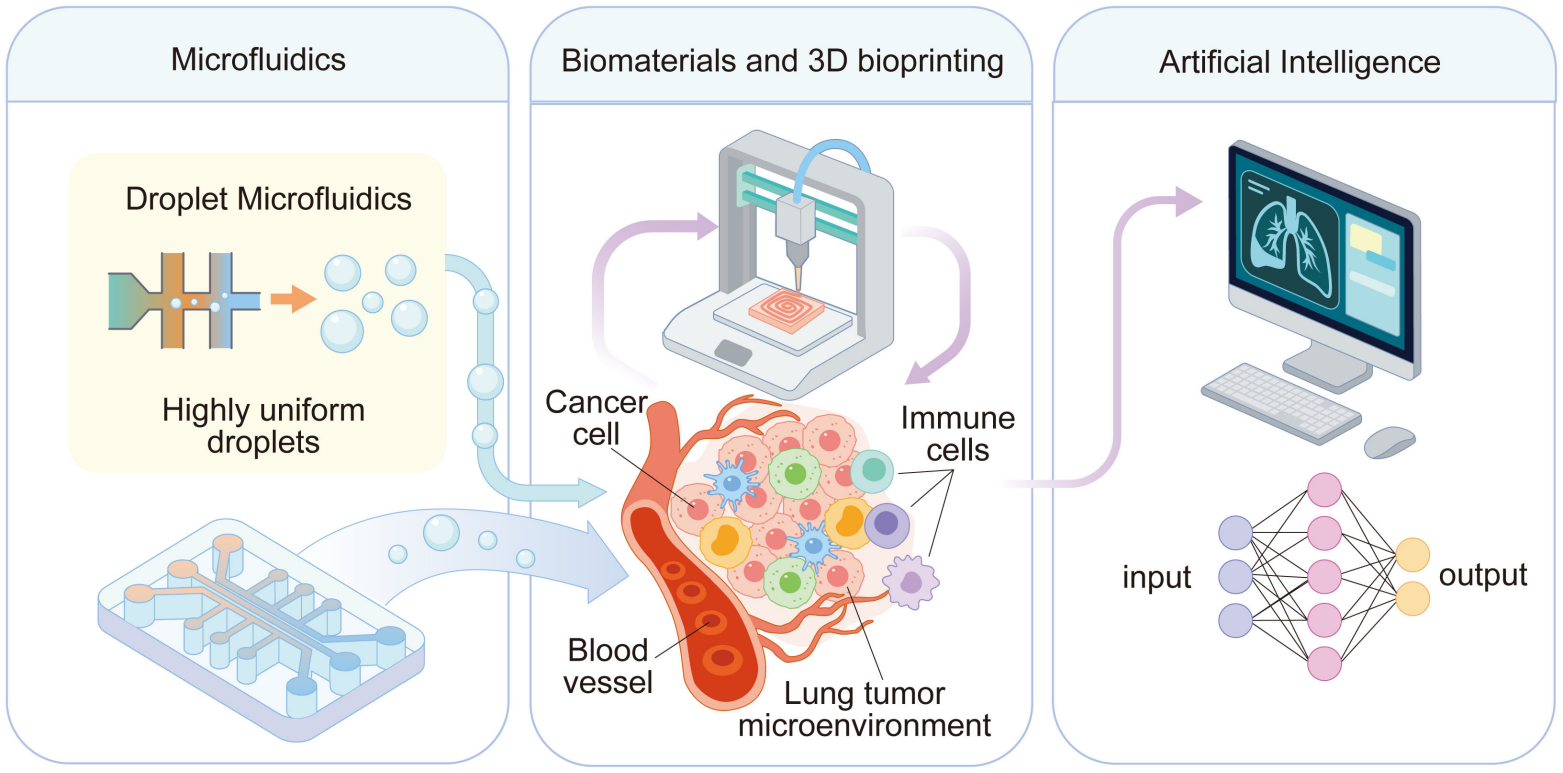
Integration of emerging engineering technologies to advance LCOs toward precision medicine. 3D, three-dimensional.

The subsequent stage of advancement will probably rely less on any single technique than on the integration of multiple modules into unified processes (**Figure 3**). Rapid generation from limited clinical specimens, controlled co-culture and exposure systems, high-content longitudinal readouts, and automated analytical pipelines collectively could turn LCOs into reproducible decision-support platforms instead of standalone experimental models. From this perspective, the translational future of LCOs lies in workflow engineering as much as in model engineering.

## 5 Evidence needed for clinical decision support

For LCOs to advance from promising translational platforms to routine clinical decision-aid tools, several layers of evidence are still required. Retrospective agreement studies are encouraging, but prospective confirmation remains essential to determine whether organoid-directed treatment selection can enhance actual patient outcomes.

Equally important is the establishment of harmonized reporting standards. Future studies should report sample source, establishment success, culture duration, tumor purity criteria, authentication strategy, assay endpoint design, and turnaround time in a more standardized manner. Without such consistency, cross-study comparison remains challenging and the field risks accumulating technically impressive but clinically non-comparable datasets.

Clinical value will also rely on how results are organized and interpreted. Physicians ultimately need actionable output, including ranked therapeutic options, resistance alerts, biomarker-supported combination strategies, and transparent confidence levels, rather than raw *ex vivo* measurements alone. This implies that the connection between organoid biology and clinical decision-making must become more structured, interpretable, and workflow-compatible.

In the future, LCO technology is anticipated to move toward more physiologically faithful and intelligent systems, potentially establishing a unified framework that connects surgical procedures, laboratory research, and clinical decision-making. The continued maturation of single-cell omics and spatial transcriptomics, when integrated with organoid platforms, will permit real-time monitoring of tumor clonal dynamics at single-cell resolution, creating chances for early detection of resistant subclones. From an engineering standpoint, next-generation organoid chips are advancing beyond single-organ modeling, as multi-organ-on-a-chip systems that include vascular, lymphatic, immune, and neural elements will allow comprehensive assessment of systemic drug metabolism, barrier function, and multi-tissue toxicity, thereby mitigating off-target effects in drug development. The incorporation of AI is further improving the analysis of complex organoid datasets. Automated extraction of micro-morphological features and molecular phenotypes may support the generation of predictive digital organoid models, enabling in silico drug testing before clinical application. As culture protocols become increasingly standardized and regulatory frameworks more clearly specified, the inclusion of organoids into clinical guidelines as validated companion diagnostics appears increasingly feasible, bringing the goal of personalized treatment based on individual tumor biology closer to practice.

## 6 Conclusion

LCOs have already progressed beyond proof-of-concept tumor models and now hold a central place in the advancement of functional precision oncology. Their greatest potential lies in connecting molecular features with dynamic phenotypic response, thus complementing genomics with directly testable functional evidence. However, broader clinical application still requires solutions to several major challenges, including normal airway epithelial contamination, culture-induced selection, incomplete reconstruction of the tumor microenvironment, limited standardization, and delays in model establishment and drug testing. Future progress will depend on integrated workflows that combine rigorous authentication, optimized culture systems, engineered matrices, microfluidic or bioprinted platforms, high-content imaging, multi-omics profiling, and AI-assisted analysis. As these challenges are gradually overcome, LCOs are increasingly evolving from specialized research models into practical decision-support tools for drug screening, biomarker discovery, and individualized treatment selection in lung cancer.
